# Innovative Options for Bone Metastasis Treatment: An Extensive Analysis on Biomaterials-Based Strategies for Orthopedic Surgeons

**DOI:** 10.3389/fbioe.2020.589964

**Published:** 2020-10-06

**Authors:** Ania Naila Guerrieri, Monica Montesi, Simone Sprio, Roberta Laranga, Laura Mercatali, Anna Tampieri, Davide Maria Donati, Enrico Lucarelli

**Affiliations:** ^1^Unit of Orthopaedic Pathology and Osteoarticular Tissue Regeneration, IRCCS Istituto Ortopedico Rizzoli, Bologna, Italy; ^2^Institute of Science and Technology for Ceramics, National Research Council, Faenza, Italy; ^3^Osteoncology and Rare Tumors Center, Istituto Scientifico Romagnolo per lo Studio e la Cura dei Tumori (IRST) IRCCS, Meldola, Italy; ^4^Third Orthopaedic and Traumatologic Clinic Prevalently Oncologic, IRCCS Istituto Ortopedico Rizzoli, Bologna, Italy

**Keywords:** bone metastasis, oncology, biomaterials, orthopedic bioimplants, 3D scaffolds, bone regeneration, smart biomaterials, patents

## Abstract

Bone is the third most frequent site of metastasis, with a particular incidence in breast and prostate cancer patients. For example, almost 70% of breast cancer patients develop several bone metastases in the late stage of the disease. Bone metastases are a challenge for clinicians and a burden for patients because they frequently cause pain and can lead to fractures. Unfortunately, current therapeutic options are in most cases only palliative and, although not curative, surgery remains the gold standard for bone metastasis treatment. Surgical intervention mostly provides the replacement of the affected bone with a bioimplant, which can be made by materials of different origins and designed through several techniques that have evolved throughout the years simultaneously with clinical needs. Several scientists and clinicians have worked to develop biomaterials with potentially successful biological and mechanical features, however, only a few of them have actually reached the scope. In this review, we extensively analyze currently available biomaterials-based strategies focusing on the newest and most innovative ideas while aiming to highlight what should be considered both a reliable choice for orthopedic surgeons and a future definitive and curative option for bone metastasis and cancer patients.

## Introduction

### Epidemiology and Clinical Background of Bone Metastasis

**FIGURE 1:**
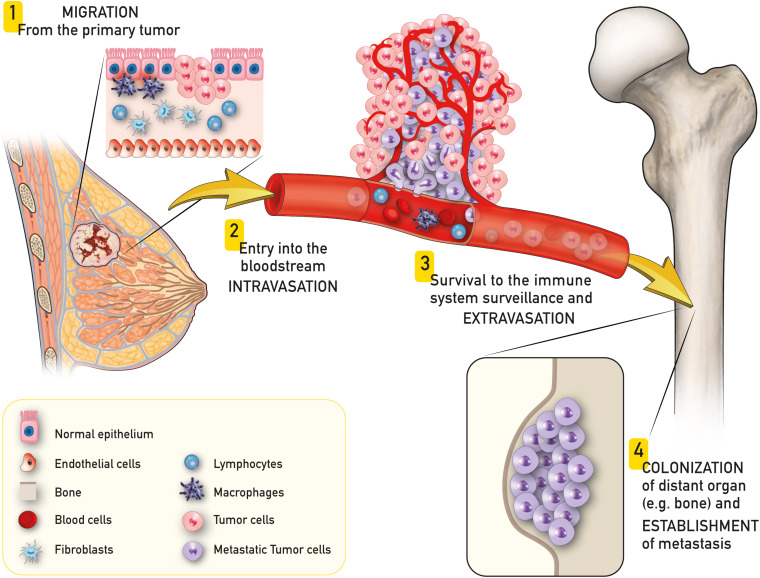
Schematic representation of breast to bone metastasis. A subgroup of cancer cells of the primary tumor (e.g., breast cancer) acquire the ability to migrate **(1)**, enter the blood stream (intravasation – **2**), survive to the immune system surveillance and exit the blood stream (extravasation – **3**) and finally spread colonizing a distant site of the body (e.g., bone – **4**).

The invasion-metastasis cascade is an extraordinary inefficient process that involves a subgroup of cancer cells of the primary tumor that acquire the ability to migrate, enter the blood stream, survive the immune system surveillance and finally spread colonizing a distant site of the body (Figure 1) (Reymond et al., 2013; Ren et al., 2015; Massagué and Obenauf, 2016; Turajlic and Swanton, 2016). Once the colony is established, metastatic cells can enter a state of dormancy, evading immune system controls to be eventually reactivated later in time (Ren et al., 2015; Sosnoski et al., 2015).

Bone is the third most frequent site of metastasis and is also the second tissue to usually require transplantation intervention after blood (Shegarfi and Reikeras, 2009; Esposito et al., 2018; Fornetti et al., 2018). It is now clear that the incidence of bone metastasis (BM) is distributed differently among primary tumor types: in particular, breast and prostate carcinomas represent the majority of tumors that metastasize to bone tissue (Harries et al., 2014; Macedo et al., 2017; Tahara et al., 2019).

As well described in literature, BM can manifest with two different kinds of lesions (Coughlin et al., 2017; Macedo et al., 2017). Specifically, breast cancers (BCs) generally causes osteolytic lesions where osteoclast activity is increased leading to disruption of tissue architecture (Yoneda, 2000; Brook et al., 2018); whereas, prostate cancer is usually linked to osteoblastic lesions in which “activated osteoblasts” alter the physiological bone formation/resorption turnover (Lin et al., 2017, 2018). More often, mixed bone lesions have been observed, probably due to a compensatory osteoblast hyperactivation as a consequence of massive bone disruption caused by osteoclasts (Tahara et al., 2019). In this scenario the interplay generated by cytokines, proteases, growth factors and receptors (e.g., EGF signaling, TGFβ, RANK-RANKL, OPG, MMPs etc.) gives rise to the so-called “vicious cycle” that involves not only cancer cells but also non-neoplastic bone cells (osteoblasts, osteoclasts, osteocytes) contributing to the break of bone homeostasis (Weilbaecher et al., 2011; Arrigoni et al., 2016; Yu and Hoffe, 2018; Dewulf et al., 2019).

BC is the leading tumor for number of cases in women which predominantly metastasize to bone; in fact, around 70% of BC patients worldwide develop BM in the advanced stages of disease (Harries et al., 2014; Tahara et al., 2019). Basing on recent studies, the main risk factors for BM development are: young age (<40 years), tumor diameter > 5 cm, high tumor grade (> BM for grade 3 tumors vs. grades 1 and 2), lobular histotype, four or more positive lymph nodes, hormonal status (e.g., positive outcome for patients with positive estrogen receptors, negative outcome for high expression of androgen receptors) and molecular profile (Lee et al., 2011; Harries et al., 2014; Feng et al., 2017; Xiao et al., 2018; Zhang et al., 2018). A recent study by Chen et al. (2017) focused on metastasis distribution among bones and found that the most frequent site affected by BM is spine, including thoracic spine (63.6%) and lumbar spine (53.8%), followed by ribs (57.5%), pelvis (54.1%), sternum (44.3%), scapula (25.1%), and femur (24.8%). BM is frequently associated with one or more skeletal related events (SREs), among them bone pain (BP), pathological fractures (PFs), spinal cord compression (SCC), and humoral hypercalcemia of malignancy (HHM). These complications are often coupled to the ones associated with surgery or radiation therapy (RT). Usually SREs appear in two thirds of patients at around 2 months to 1 year from the diagnosis, ultimately affecting patients’ (and patients’ families) quality of life (QoL) (Harries et al., 2014; Brook et al., 2018; Zhang et al., 2018; Tahara et al., 2019). As previously stated, BC cells usually show organotropism for bone tissue, making this an aspect of primary importance for further investigations. The main clinical objective remains to prevent the progress of cancer to more advanced stages, where alternative strategies to surgery and chemotherapy, are always less frequent and effective.

### Why Do Cancer Cells Metastasize the Bone? The Molecular Insights of BM

The first observation that cancer cells from the primary tumor are “primed” to colonize a specific tissue comes from the “seed and soil” theory of Stephen Paget declared in 1889 (Paget, 1889). In his theory, Paget compared cancer cells leaving the primary tumor to seeds that germinate only when sowed in a suitable soil (target organ/tissue). In fact, studies showed that the determinant factors for the metastatic site involve its micro-environmental features and the biological predisposition of cancer cells (Yoneda, 2000; Smith and Kang, 2017; Owen and Parker, 2019; Montagner and Dupont, 2020). Firstly, bone can be considered a fertile and productive soil due to its richness in growth factors and cytokines (e.g., TGF-β, IGF-1/2, BMPs, IL11, etc.) produced by osteoblasts, stored in bone matrix, and released during physiological bone disruption becoming bioavailable for cancer cells (Florencio-Silva et al., 2015; Owen and Parker, 2019). In addition, the primary tumor sends systemic signals to set and prepare suitable soil for the metastasis, the so called premetastatic niche (PMN), where stromal cells are also recruited to host cancer cells (Owen and Parker, 2019; Sowder and Johnson, 2019).

Secondly, in recent years, different studies investigating the molecular signature of bone-seeking BC cells have been published (Byler et al., 2014; Fazilaty and Mehdipour, 2014; Bahrami et al., 2016; Savci-Heijink et al., 2016). Mainly, several works have highlighted the role of TGF-β in the early stages of BC metastasis, demonstrating that it stimulates the expansion of pro-tumorigenic myeloid cells that participate in tumor progression; in fact, the inhibition of this pathway resulted in reduced osteolytic lesions and incidence of BM (van’t Veer et al., 2002; Meng et al., 2016; Buenrostro et al., 2018). Another important player in BM induction is the transcription factor Runx2; in fact, its expression in BC cells is related to bone colonization through the involvement of several pathways that stimulate cellular invasive and aggressive phenotype (e.g., metalloproteinases) (Li et al., 2016; Vishal et al., 2017). In the context of the connection between inflammation and cancer, Holen et al. (2016) discussed the role of the proinflammatory cytokine IL-1B overexpression in the development of BM, previously observed in different cancer types. With a particular focus on BC, the authors developed a mouse model of metastasis, where mice were injected with MDA-MB-231 IV, BC bone-seeking cancer cells, and found that the pharmaceutical inhibition of IL-1B bond to its receptor IL-1R, leads to a reduction in metastatic tumor growth and cellular proliferation. This work further demonstrated the determinant role of IL-1B in BM onset, therefore highlighting it as a new potential biomarker for BC progression and a therapeutic target (Holen et al., 2016). Lately, Bellanger et al. (2017) identified the *ZNF217* oncogene as a critical player in BM development and an early biomarker, being up-regulated at the mRNA level in BC primary tumors that usually metastasize to bone. The authors demonstrated that *ZNF217* overexpression is also specific for bone-only metastasis. In the same work, Bellanger et al. (2017) generated a *ZNF217* stable overexpressing cell line confirming the acquisition of an aggressive phenotype *in vivo* with the presence of multiple and severe osteolytic lesions, probably linked to the dysregulation of the expression of a subset of genes involved in the epithelial-mesenchymal transition (EMT) and bone morphogenetic proteins (BMPs) pathways. Interestingly, some of these genes have also been found related to BM tropism in two previously published studies (Kudo et al., 2003; Bellahcène et al., 2007; Bellanger et al., 2017). Another interesting and recently proposed biomarker of BM development from BC is leptin; in fact, it is associated with the induction of BC cells metastasis to bone tissue through the activation of the SDF-1/CXCR4 signaling and the induction of the EMT (Duan et al., 2020).

Beyond the role of the above mentioned (but not limited to) single genes and proteins in BM onset (Sgroi, 2009; Byler et al., 2014; Holen et al., 2016; Bellanger et al., 2017; Jin et al., 2018; Duan et al., 2020), different research groups have analyzed the whole molecular profile of primary tumors from patients with BM and have identified a so called “molecular signature” (van’t Veer et al., 2002; Kang et al., 2003; Woelfle et al., 2003; Fazilaty and Mehdipour, 2014; Bahrami et al., 2016; Lefebvre et al., 2016; Savci-Heijink et al., 2016). Focusing on BC, Savci-Heijink et al. (2016) published the largest study to date on the association between gene expression profiling in primary BC and BM finding a 15-gene signature that could potentially help both in BM early diagnosis or prevention and in the therapeutic approach for this kind of disease. In their work, the authors compared their findings with another gene signature previously published by Kang et al. (2003), where samples from BM (and not primary tumors) were analyzed. The apparent discrepancy between the two gene signature sets is indeed proof of the fact that cancer cells deriving from the primary tumor have a specific molecular profile that induces bone tropism; once reached the metastatic site, the expression of different genes is changed depending on the cellular needs to survive in the host tissue. Furthermore, these results highlight the importance of selecting samples depending on the objective of the study and that a combination of different studies may help in understanding the molecular changes that occur through the metastatic process.

In this complex scenario, bioinformatics comes to help in the identification of genes linked to BC progression and BM onset. For example, Cai et al. (2017) built up a computational method based on human protein-protein interactions and to test the accuracy of their method, they analyzed the previously validated genes related to BC and BM onset. The authors constructed an algorithm that allowed to find other eighteen genes which are involved in the metastatic cascade from breast to bone (Cai et al., 2017).

More recently, Li J.-N. et al. (2019)set up another useful bioinformatic tool based on a minimal driver gene set in gene dependency network to distinguish patients that will develop BM from low risk ones. Specifically, the tool exploits an algorithm based on gene dependency network, with the hypothesis that the relation between one gene and the risk of BM is potentially influenced by another gene. In addition, Li J.-N. et al. (2019) found that their signature is specific to BM, excluding tropism for other organs.

Further studies comprised of larger sample sizes are necessary on this topic as it is clear that molecular signatures may represent a useful tool in intervention at early stages to prevent BM onset and in parallel to finding the best medical strategy possible, such as target therapy (Dewulf et al., 2019). In addition, other complex mechanisms are involved in BM establishment and their role needs to be more considered and investigated; among these micro-RNAs expression, tumor-released exosomes, epigenetic and translational modifications are worth to be cited. To study more in depth these aspects the readers are referred to the following references (Byler et al., 2014; Hoshino et al., 2015; Zoni and van der Pluijm, 2016; Li F. et al., 2019).

### Treatment Strategies for BM

The importance of understanding the natural history of BM and preventing its onset is based on the fact that, nowadays, treatment options are limited and often inefficient to be completely curative; in fact, treatments aim still remains to prolong patient survival and ameliorate their QoL (Sambi et al., 2013). Currently, applicable systemic therapies include chemotherapy and bone-targeted therapies (BTTs) as bisphosphonates and other antiresorptive molecules such as anti-RANK-RANKL inhibitors (Brook et al., 2018; Soeharno et al., 2018). A recent multi-country cross-sectional study carried out by Body et al. (2019) on the management of patients with prostate cancer shows that the majority of BM patients (74%) are treated with BTTs, such as zoledronic acid (bisphosphonate) and denosumab (human monoclonal antibody against RANKL) especially if they are followed by an oncologist instead of a urologist. Other types of biological drugs direct on specific targets involved in BM formation such as anti-CXCR4 and Cathepsin-k Inhibitors have been investigated (Body et al., 2019). Apart from systemic therapeutic approaches, some local interventions are used: for example, radiotherapy with radioisotopes with affinity to bone (e.g., strontium-89, RAD001, Lu177-PMSA) represents another palliative option for BM intervention when metastases are a few and easily confinable (Sambi et al., 2013; Bianchi et al., 2016; Errani et al., 2019). Recently, electrochemotherapy has also been used to treat BM, and the results of a phase II clinical study confirm that this treatment controls tumor growth and pain (Bianchi et al., 2016).

Finally, surgery is performed in case of risk of fracture especially when BM affects the femur, humerus, spine and hip (Errani et al., 2017, 2019; Macedo et al., 2017). A surgical intervention provides first, the removal of metastatic tissue, trying to spare the largest quantity of healthy tissue possible, and second, the replacement of bone parts with prostheses or bioimplants (Errani et al., 2017). However, in some cases bone damage caused by tissue disruption during the procedure could represent a severe cause of disability (Sambi et al., 2013).

Bioimplants used for the replacement of affected bone can be made of different materials; each current available class of materials has different properties from both the biological and mechanical standpoint. All of these features need to be carefully considered and studied in order to avoid implant rejection and/or infection, and the consequential additional surgery for patients. A meticulous upstream design of the implant represents a successful strategy to avoid further inconvenience and pain to patients, as well as additional costs for healthcare systems. The main objective of this review is to analyze the most current promising research approaches in the field of biomaterials-based strategies for metastatic bone treatment, highlighting the ones that can be considered both a reliable choice for orthopedic surgeons and a future definitive and curative option for BM and cancer patients. At the end, we will discuss some interesting patents that, in our opinion, could represent in the near future an important clinical innovation for the replacement of damaged bone tissue.

## Biomaterials for Bone Tissue Applications

In the case of BM and bone tumors requiring the ablation of diseased parts, the recourse to bone substitutes can be mandatory. Nowadays the best material for bone reconstruction still remains the autologous one, usually obtained from non-loadbearing skeletal sites (e.g., iliac crest, ribs, chin, parts of the skull) (Baino, 2017). Although autografts imply optimal biocompatibility, no risk of immunogenic response, and high regenerative properties, there are some limits to their usage in clinical practice because of restricted availability of the tissue, as well as enhanced patient morbidity with possible clinical complications due to the process of harvesting the patient’s own tissue (Baumhauer et al., 2014; Baino, 2017). Alternative methods rely on allografts, namely use of tissue from other individuals/cadavers or from animals. This, however, raises concerns in terms of biocompatibility, chances of zoonosis transmission, and involve significantly high handling costs (e.g., tissue bank management). For these reasons, the use of synthetic biomaterials is currently considered as the best option. Throughout the years, the demand for bone substitute materials has increased, principally due to the aging population and the need to guarantee the preservation of QoL standards to patients (Prasad et al., 2017).

The fundamental concept that should be kept in mind when approaching or considering the design of a biomaterial is that bone is basically a composite material. In fact, it is made of an inorganic part constituted of carbonated calcium deficient hydroxyapatite (HA) nanocrystals (25–50 nm of diameter), nucleated on a collagenous macromolecular matrix which, by supramolecular organization from the nano to the macroscale, is subjected to maturation and remodeling to assume a complex 3D hierarchical porous structure (Reznikov et al., 2014; Vallet-Regí and Salinas, 2019). Furthermore, this complex architecture is key for its astonishing mechanical performance, thus biomaterials should be designed in order to elicit specific cell behavior leading to the formation and structural organization of new bone.

Considering the challenges in BM treatment and the clinical need of new approaches, during the past several years, scientists have directed their efforts to create innovative biomaterials with increasing ability to interact with host tissue and to be suitable for use in both tumor therapy and bone tissue regeneration.

### First Generation Biomaterials

Historically, the development of bone biomaterials initiated in the 1960s with inert ceramic or metallic substitutes (the so called first generation), with the purpose of mechanical sustention at the bone defect site. Examples of these types of materials are ceramics such as alumina (Al_2_O_3_) and zirconia (ZrO_2_), used in clinical practice since 1960 throughout the1980s in femoral head implants, hip balls and also total knee substitutes (Smith, 1963; Boutin, 1972; Oonishi et al., 1981). Such ceramic materials are hard yet brittle, although zirconia is characterized by relatively low elastic modulus (similar to that of cortical bone) combined with good strength and durability that, for example, make it more suitable for femoral head implants than alumina (Bose et al., 2017; Vallet-Regí and Salinas, 2019). The major limits linked to the use of zirconia implants are related to the formation of chemically stable debris that can cause unhealthy biological responses, together with the eventual presence of radioactivity (^235^U traces) (Vallet-Regí and Salinas, 2019).

In addition to ceramics, metals have, in general, a wider range of application in orthopedics, from small components, such as pins, screws, and plates, to larger load-bearing implants, total joint, and total knee replacements (Ghosh et al., 2018; Minnath, 2018). This is due to relatively high elastic moduli, plasticity and yield points that together make them generally suitable for load-bearing applications, even in the case of series of load-unload cycles; moreover, with appropriate surface treatment, alloys combination and/or production method, metal implants can reach good resistance in all body compartments (Ghosh et al., 2018). Likewise bio-ceramics, metallic based biomaterials have “evolved” during the years together with material science, surface chemistry and biological/clinical evidences; in this respect, bioinert metals such as stainless steel (SSt), titanium (Ti), and cobalt-chromium alloys (Co-Cr) underwent chemical surface modifications or coating procedures to improve osteo-biologic properties (Asri et al., 2017; Dehghanghadikolaei and Fotovvati, 2019).

In parallel, a huge variety of polymers have been tested as bone substitutes (Kashirina et al., 2019). However, only a few of them are suitable to be used as unique constituent of a final implant, such as poly(methyl methacrylate) (PMMA), polyaryletherketones (PAEK) and ultrahigh-molecular-weight-polyethylene (UHMWPE).

PMMA is a thermoplastic polymer, characterized by ease of manufacturing and handling, good biocompatibility, appropriate mechanical strength and elastic modulus. However, its use raises several health concerns (Bose et al., 2017; Gohil et al., 2017; Bistolfi et al., 2019). For these reasons, even if PMMA has been widely used in spinal vertebroplasty or as bone filler in primary tumor or metastatic patients, nowadays new applications for its use have been developed almost exclusively in composite materials and in 3D-printing of customized bioimplants (Kawashita et al., 2010; Han et al., 2011; Teo et al., 2016; Robo et al., 2018).

The term “polyaryletherketones” (PAEK) is referred to a family of semi-crystalline thermoplastic compounds among which the most commonly investigated for orthopedic applications are poly(etheretherketone) (PEEK) and poly(etherketoneketone) (PEKK). Generally, these polymers are classified as high-performance polymers (HPPs) and are proposed as an alternative to metallic implants due to particular mechanical and chemical properties (Wiesli and Özcan, 2015; Gohil et al., 2017).

UHMWPE is one of the most frequently used polymers in orthopedic applications and still represents a valid alternative to pure metals or their alloys, particularly when embedded in composite materials, due to relative lower costs, ease of manufacturing, and availability. However, serious problems related to UHMWPE are its oxidative degradation, with consequent release of free radicals around the implant, and short-term durability; these limits are currently faced by surface chemical modification or through reinforcement procedures such as blending with other materials [e.g., carbon nanotubes (CNTs), HA, graphene oxide (GO) etc.] (Patil et al., 2020).

### Second Generation Biomaterials

Both metals and polymers show quite different features from target bone tissues implying the occurrence of adverse reactions that, even in the short term, can jeopardize the healing process. Furthermore, we need to consider that such a device offering merely a physical sustention, cannot guarantee bone healing and regeneration. To address this issue, the approach taken by material scientist in the 1980s was dedicated to developing bioactive scaffolds, which, at the time were known as second generation materials, where bone-like composition and porous structures were able to promote new bone formation and osteointegration. Typical examples are calcium phosphates such as HA, calcium sulfates and bioglasses (BGs); in particular, synthetic HA is most commonly used due to its similarity in Ca/P molar ratio and structure to bone apatite (Baino, 2017; Vallet-Regí and Salinas, 2019). Synthetic HA is biocompatible, bioactive and osteoconductive, specifically when partially substituted with carbonate ions and when used in its nanostructured form with a 4–8 wt% CO_3_^2–^ ions (in the PO_4_^3–^ sublattice) (Vallet-Regí and Salinas, 2019). It can also be included in mixtures with β-tricalcium phosphate, β-Ca_3_(PO_4_)_2_ (β-TCP), showing an interesting combination of HA stability and higher resorbability of β-TCP alone. This material is osteoinductive and osteopromoting due to the release of Ca^2+^ and PO_4_^3–^ ions that stimulate new bone formation. It also has the advantage of being used in different formulations, as powder-coating, bulk, injectable paste or, more recently, 3D-printed for complex bone defects applications (Fellah et al., 2008; Sánchez-Salcedo et al., 2008; Castilho et al., 2014; Kitayama et al., 2016; Vallet-Regí and Salinas, 2019).

BGs are obtained mainly as granules for bone defect filling, but at the time of their first development, they were considered as osteoinductive materials able to bind with bone without the formation of fibrous capsules around it (Jones, 2013; Jones et al., 2016; Baino, 2017). In fact, their high reactivity with body fluids leads to the formation of a surface hydroxycarbonateapatite (HCA) nanocrystalline layer that accelerates bone tissue growing and matrix deposition (Baino, 2017; Vallet-Regí and Salinas, 2019). In general, glasses are not common in the orthopedic field because of their poor mechanical properties, namely brittleness, low bending strength, and fracture toughness, which cannot meet the needs of large bone tissue applications (Jones et al., 2016; Baino, 2017).

### Third Generation Biomaterials

The further advancement during the years in biomaterial development, or rather, the definition of third generation biomaterials, is related to the ambition to completely regenerate critical size bone defects. Hence, the aim of scientists has been to develop bone devices able to reabsorb during new bone formation, with the purpose to allow complete replacement of the scaffold with newly formed bone tissue, thus achieving enhanced physical stabilization and improved bone functionality. These biodegradable materials, particularly calcium phosphates and mesoporous BGs, are generally no longer used as monolithic or bulk phases but rather as porous scaffolds as a way to further stimulate interactions with bone tissue and its self-healing properties (Huang, 2017; Vallet-Regí and Salinas, 2019). Fundamental aspects in this group of ceramics are firstly their “smart” capacity to adapt and modify their properties to the changing implant site environment, and secondly, a diverse handling method, mostly at room temperature, that allow the inclusion and preservation of bioactive molecules (drugs/antibiotics, growth factors, magnetic particles for thermal cancer therapy) (Vallet-Regi et al., 2012; Comesaña et al., 2015; Koju et al., 2018; Vallet-Regí and Salinas, 2019). With the purpose to obtain enhanced therapeutic effectiveness, these materials were therefore tested in many studies as drug carriers or as gene delivery systems (Ruiz-Hernández et al., 2008; Liu et al., 2009; Vallet-Regí and Ruiz-Hernández, 2011; Wu and Chang, 2014; Gao et al., 2015; Santos et al., 2017; Köse et al., 2018; Afewerki et al., 2020). To better resemble natural bone apatite and interact with neighbor tissues, synthetic HA can include several diverse ions in place of Ca^2+^, PO_4_^3–^, or OH^–^, e.g., Na^+^, K^+^, Mg^2+,^ Sr^2+^, Zn^2+^, Cl^–^, F^–^, HPO_4_^2–^ (Kim et al., 1998; Vallet-Regí and Arcos, 2005; Landi et al., 2007, 2008; Kolmas et al., 2011; Bornapour et al., 2013; Montesi et al., 2017; Arcos and Vallet-Regí, 2020). The incorporation of these ions in the HA structure limits the crystal growth and alters surface properties, thus enhancing its bioactivity and integrability with tissues; additionally, it also helps bone tissue self-healing and regeneration through the stimulation of osteoblasts and bone resident cells to form new bone when released during implant degradation (Vallet-Regí and Salinas, 2019). For all these reasons, HA has been used not only as the pure constituent of implants but also specifically as nano-HA for coatings e.g., in Ti alloys, Ti_6_Al_4_V, and in sol-gel preparations, where bioactive responses and biocompatibility is far higher than pure HA (Khor et al., 2004; Kulpetchdara et al., 2016; Sánchez-Salcedo et al., 2016; Zhou et al., 2016; Domínguez-Trujillo et al., 2018; Izquierdo-Barba et al., 2019; Lapaj et al., 2019; Robertson et al., 2019; Vallet-Regí and Salinas, 2019; Arcos and Vallet-Regí, 2020).

Calcium phosphates are also the base of inorganic bone cements with good osteoinductive potential (Dorozhkin, 2008; Boanini et al., 2010; Bohner, 2010; Schumacher et al., 2013; Sprio et al., 2016a). An important advantage of calcium phosphate cements is the ability of self-hardening by dissolution/reprecipitation processes occurring *in vivo* at body temperature that yields the transformation of metastable calcium phosphates into calcium-deficient HA with acicular morphology. The possibility of doping with bioactive ions and also the implementation with natural polymers makes it possible to obtain self-consolidating apatite scaffolds characterized by high bioactivity induced by biomimetic composition and nanostructure, diffuse microporosity, and good mechanical strength. Critical aspects of this kind of cement are injectability, setting time, which must be compliant with the timing of the clinical procedure, and the exposure of wide open porosity, while maintaining effective mechanical properties (Vallet-Regí and González-Calbet, 2004; Zhang et al., 2014; Hughes et al., 2015; Vallet-Regí and Salinas, 2019).

Even if metals are rarely biodegradable, there are some that possess this feature and among them, magnesium (Mg), iron (Fe), and zinc (Zi) are the most used for orthopedic implants (Prasad et al., 2017). Generally, these metals show good biocompatibility because of the non-toxic nature of released ions and sufficient strength to support bone tissue ingrowth (Heiden et al., 2015; Prasad et al., 2017). Among these biodegradable metals, Mg is particularly interesting; in fact, it shows high tensile strength, relatively low Young’s elastic modulus and relatively low density compared to other metals, and similar properties to that of cortical bone, potentially reducing stress shielding related risks, typical of titanium (Ti) and other high-strength metals (Shayesteh Moghaddam et al., 2016; Prasad et al., 2017; Zhao et al., 2017; Minnath, 2018). Thanks to these mechanical properties, Mg is mostly used to build bone fixation devices (e.g., pins, screws, rods and plates) and more recently, load-bearing devices in combination with other metals such as SSt or Ti. The degradation kinetics of Mg implants can be adjusted through the development of alloys with other non-toxic elements, like calcium (Ca) and zirconium (Zr), or by way of mechanical and chemical surface treatments (Shayesteh Moghaddam et al., 2016; Prasad et al., 2017; Zhao et al., 2017; Minnath, 2018; Bordbar-Khiabani et al., 2019). The released Mg^2+^ ions positively stimulate osteoblasts and other bone cells activities and proliferation, being beneficial for new bone tissue development and implant fate (Zhao et al., 2017; Ghosh et al., 2018). More recently, metals are used to build 3D porous scaffolds and implants that better allow cellular adhesion, proliferation and differentiation leading to new bone formation *in vivo* (Shah et al., 2016; Fraser et al., 2019; Zadpoor, 2019); moreover, metal implants surface has been functionalized with bioactive molecules or drugs (Su et al., 2018, 2019).

Contextually, biodegradable polymers were largely investigated, with particular respect to poly(glycolic acid) (PGA), poly(lactic acid) (PLA), poly(lactic- co-glycolic acid) (PLGA), poly(ε-caprolactone) (PLC) and related copolymers (Puppi et al., 2010; Zhao et al., 2017). PGA, PLA, and PLGA are poly(α-hydroxyacids) extensively studied for bone tissue engineering due to their relatively easy manufacturing and their biodegradability, as well as physical and mechanical properties, that are tunable by modification of their molecular weight or by creating composites (Puppi et al., 2010). However, their degradation products are usually acidic monomers that easily provoke alterations of the physiologic pH around the implant when released in high amounts, thus leading to inflammatory responses of varying entity (Puppi et al., 2010; Gohil et al., 2017). The major limit of poly(α-hydroxyacids) and PCL is their hydrophobicity that leads to poor wettability and consequently scarce cellular and molecular interactions. Chemical surface modification, such as silanes treatments, are usually exploited in order to improve their surface reactivity and biocompatibility (Puppi et al., 2010; Jaidev and Chatterjee, 2019), as well as surface functionalization with bioactive molecules, such as rhBMP2, BMP7 or RGD peptides, aiming to significantly improve cells adhesion, proliferation and new bone formation *in vitro* and *in vivo* (Rahman et al., 2014; Kim et al., 2015; Shin et al., 2015). In particular, it has been recently shown that porous PLGA scaffolds loaded with both rhBMP2 and BTTs, e.g., zoledronic acid and IkappaB kinase (IKK)-inhibitors promoted both bone regeneration and antiresorptive activity in order to stimulate healing of critical bone defects in rats (Yu et al., 2014). The promising results of this work open the possibility to design new medicated scaffolds with both pro-regenerative molecules and antineoplastic agents to better treat BM patients. Another issue related to polyesters, PCL and similar compounds (e.g., polyanhydrides, polyfumarates, polycarbonates, polydioxanone) is related to their poor mechanical properties, so that a main approach is to blend them with inorganic reinforcing phases or fibers, also resulting in the improving of osteobiological properties (Puppi et al., 2010; Yao et al., 2017; Donnaloja et al., 2020

### Fourth Generation and Smart Biomaterials as a New Strategy for BM and Cancer Therapy

The current increasing need to achieve bone regeneration along with treating concurrent pathologies afflicting bone metabolism such as osteolytic/osteoblastic metastasis, primary cancers, or osteoporosis, is motivating material scientists to develop smart bio-devices endowed with multiple bio-functionalities. These fourth generation biomaterials are therefore able to promote tissue regeneration while attempting to equilibrate cell impairment and to contrast infections. The main purpose is to reproduce as best as possible all the features of the extra cellular matrix (ECM) by associating biomimetic composition, nanostructure and 3D architecture to promote natural cell metabolism and the activation of the appropriate molecular pathways. In this respect, there is growing interest on the effect of electric fields on cells, along with the prevention of bacterial proliferation. Therefore, recent approaches based on bioelectricity attempted to modulate cells’ fate by the delivery of bioelectric signals using electrophysiology or by activation of electric charges, as induced by the inherent chemistry and nanostructure of biomaterials (Sachot et al., 2015; Ning et al., 2016). In this regard, it was recently discovered that specific ion doping in the structure of HA phase enhances the segregation of various ionic species at the surface, which is responsible for enhanced osteogenic and antibacterial ability (Sprio et al., 2019). Moreover, other approaches rely on the use of composites where the electric conductivity can be improved by incorporation of conductive phases such as CNTs (Gonçalves et al., 2016). The use of composites can enlarge the applicability of 4D-printing technologies, with the aim to realize scaffolds that can adapt and grow with the host, paving the way to interesting applications in younger patients as well (Invernizzi et al., 2019; Wan et al., 2020).

Focusing on the achievement of close mimicry with natural bone tissue, the use of collagen and other natural polymers is of increasing interest in material science. Such polymers can be obtained both from animal sources (e.g., collagen, chitosan) and plant sources (e.g., alginates, cellulose, silk fibroin); altogether they represent a valid option among biomaterials because of low manufacturing and management costs, ease of supply and renewability, biodegradability, biomimicry and biocompatibility. However, the limits of natural polymers are not negligible and include the following: rejection and immunogenic response, disease transmission, fast degradation rate and poor mechanical properties that preclude their applications as pure constituent in orthopedic implants (Puppi et al., 2010; Bose et al., 2017; Donnaloja et al., 2020). Since collagen is the major constituent of the organic part of bone, it is particularly interesting and has been used alone or in composites in various forms such as fibers, foams and sponges. It has also been widely used as a bioactive molecule carrier (Puppi et al., 2010; Cunniffe and O’Brien, 2011; Ige et al., 2012; Gohil et al., 2017; Donnaloja et al., 2020). Nevertheless, collagen alone does not mimic the complex composition of bone tissue; the unique properties of bone are strictly related to its complex 3D structure and to the chemical interaction of its mineral nuclei with the collagenous matrix. In fact, during bone formation, collagen molecules expose their charged sites for the heterogeneous nucleation of the bone mineral nuclei, whose growth and organization, in turn influencing cell chemotaxis, is regulated by various control mechanisms acting from the molecular to the nano-scale (Tampieri et al., 2011). In the attempt to reproduce the complex features of bone tissue, conventional fabrication methods based on high temperature processing usually fail to achieve 3D scaffolds retaining the bioactive composition and nanostructure of the mineral bone. In this regard, recent nature-inspired approaches allowed to obtain the heterogeneous nucleation of ion-doped HA nuclei onto collagen fibrils during their supramolecular, pH-driven, assembling into highly porous, hybrid 3D scaffolds; the so obtained structures are able to deform elastically in physiological media, thus showing remarkable adaptability to tissue defects and ability of metabolic resorption that can be tailored by appropriate cross-linking processes (Sprio et al., 2016b; Krishnakumar et al., 2018). These materials demonstrated *in vitro* and *in vivo* osteoinductivity, highlighting both osteogenic and angiogenic potentials (Calabrese et al., 2016a,b). Moreover, in a recent clinical study involving 41 patients, such hybrid scaffolds demonstrated an ability to provide good arthrodesis to obtain long spinal fusion in the treatment of adult scoliosis (Giorgi et al., 2017).

Currently, hybrid scaffolds with superparamagnetic properties were obtained by inducing the nucleation of Fe-doped HA, a recently developed bioactive nanophase where magnetic properties take origin from the particular positioning of Fe^2+^/Fe^3+^ ions in the HA structure (Tampieri et al., 2014; Iannotti et al., 2017). Fe-HA nanoparticles have already shown the ability to modulate the release of the anticancer drug doxorubicin under an applied low-frequency pulsed electromagnetic field (PEMF) (Iafisco et al., 2016). This achievement opens to clinical applications where external magnetic fields will remotely activate specific bio-functionalities such as direct magnetic stimulation of bone cells as a boosting of the endogenous regenerative potential, particularly indicated for the elderly, or on-demand drug release for personalized medicine (Tampieri et al., 2019b).

In a different, more recent approach, biomorphic scaffolds made of Mg^2+^/Sr^2+^-doped HA were obtained through a sequence of heterogeneous reactions able to transform natural woods into ion-doped HA, while retaining the multi-scale complex architecture of the original wood template which strongly mimic the osteon structure of the compact bone (Tampieri et al., 2019a; Sprio et al., 2020). The biomorphic HA scaffold showed great ability to modulate the stem cells’ fate and to enhance the cross-talk between endothelial and mesenchymal stem cells (MSCs), as induced by the pore architecture mimicking the lymphatic network of trees. In this sense, this HA-based scaffold is promising for the stimulation of both bone and vascular regeneration, a parameter that is usually neglected. Furthermore, biomorphic HA scaffolds show unusual mechanical properties for a ceramic, i.e., higher tensile than compression strength, bone-like Young’s modulus, as well as high durability and ductility. These features are permitted by the multi-scale structural hierarchy that helps to prevent sudden fracture, typical of pure ceramic materials, and to show damage-tolerant mechanical behavior complying with the natural bone performance (Bigoni et al., 2020).

Another promising strategy relies on organic-inorganic hybrid materials where the inorganic part takes advantage on the ions exchange potential of the surface silanol groups of mesoporous BGs (or other oxides groups, e.g., on calcium phosphate cements) and the organic part is usually composed by biopolymers. This kind of composite materials have a great impact on the improvement of mechanical properties in respect of pure/single constituent ones: for example, silica-bases “star-gels” organized as an organic core with inorganic flexible arms, reached characteristics between glasses and rubbers similar to that of human tibia, making them a suitable option for long bone implants (Michalczyk et al., 1996; Manzano et al., 2006; Vallet-Regí and Salinas, 2019).

With a direct focus on BM and cancer, matching dual function, therapy and regeneration, recently, Liu and collaborators proposed a bifunctional element (Cu, Fe, Mn, Co)-doped bioactive glass-ceramics scaffold fabricated by combining the sol-gel method and a 3D-printing technique and demonstrated that the incorporation of these elements into the biomaterial endowed the scaffolds with excellent photothermal properties, tuned by the laser irradiation under ultra-low power density application. The high temperature generated by these scaffolds effectively killed tumor cells *in vitro* and significantly inhibited tumor growth *in vivo*. Furthermore, the bioactive elements doped scaffolds significantly promoted osteogenic differentiation of bone-forming cells (Liu et al., 2018). The simultaneous ability of tumor therapy and bone regeneration has been also explored in other studies of the same group in 2016 and 2018, where the authors proposed a bifunctional graphene oxide (GO)-modified β-tricalcium phosphate (GO-TCP) composite scaffold and BG-CFS scaffolds via *in situ* growing CuFeSe2 (CFS) nanocrystals on the strut surface of BG material, combining a high photothermal effect with significantly improved bone-forming ability (Ma et al., 2016; Dang et al., 2018).

In the last few years, researchers have shown great attention to the use of magnetic nanoparticles for hyperthermia upon application of an external, alternating magnetic field. John et al. (2017) developed a method for producing a 3D macroporous scaffold with controlled size and shape of pores decorated by HA and carbonate hydroxyapatite (CHA) and doped with spinel-like MgFe_2_O_4_ magnetic nanoparticles for potential application in hyperthermal therapy that may also positively affect bone tissue regeneration. Another interesting approach to treat BM and tumors by hyperthermia is based on the utilization of a polymeric matrix embedding magnetic fillers (Miola et al., 2019). In this regard, a composite bone cement obtained by incorporation of ferrimagnetic and bioactive glass-ceramic micro-particles in the PMMA polymeric matrix in different amounts has been proposed (Bretcanu et al., 2006). This material is able to generate heat under an external alternating magnetic field sustaining also osteoblast viability and interaction with the biomaterial itself (Verné et al., 2015).

Moving the attention to metallic biomaterials, between Ti alloys nitinol (NiTi) is an interesting one. NiTi is classified as a smart material with superelastic properties, provided with plastic deformation at low temperature, that allows shape memory features (Jones et al., 2017; Minnath, 2018; Sinha et al., 2018). NiTi has great potential to be used in a large variety of applications preserving the high biocompatibility of Ti, and unlike other Ni containing alloys it is not subjected to excessive Ni toxic ions release (Minnath, 2018; Sinha et al., 2018). Although, poor clinical evidences of NiTi use in orthopedic clinics are currently available, an interesting feature of porous NiTi implants, that could be used to mimic trabecular bone, is their pump-like and capillary behavior that canalize fluids, cells and new growing vessels inside the material’s pores, guaranteeing a greater integration of the whole implant and a contemporary stimulated growth of new bone tissue. This characteristic make porous NiTi also bioactive, in respect of bulk NiTi or other Ti alloys (Shayesteh Moghaddam et al., 2016; Gorgin Karaji et al., 2017). Moreover, porous NiTi could be a valid alternative to mitigate stress shielding caused by the excessive stiffness of other Ti alloys plates used in bone fixation applications. Bulk NiTi has the lowest stiffness between other Ti alloys (around 36–68 GPa) that can be further reduced near to that of cortical bone (10–31.2 GPa) introducing porosity. In this way it is possible to obtain stiffness-matched NiTi that showed promising results in mandibular surgery and may, therefore, represent an alternative also for other orthopedic applications in metastatic bone treatment (Niinomi and Nakai, 2011; Amerinatanzi et al., 2016; Jahadakbar et al., 2020). On this regard, a recent published patent by Fonte et al. (2018) provides information on the manufacturing of a NiTi based porous orthopedic implant produced via an injection molding process. The so obtained material has potential applications in bone defect repair as bone void filler and augmentation device, to fill gaps between the medical implant and adjacent bone generating a dynamic and osteoconductive interface, or generally to increase osteoconductive potential of a Ti/Ti alloys-based implants (Fonte et al., 2018). Basing on that further research and *in vivo* studies may be useful to really understand porous NiTi potentials in BM interventions.

Thanks to the possibility of the concept of “tumor-specific design,” the last several years have seen significant improvements in nano-sized biomaterials development and their use as therapeutic, diagnostic tools, and imaging agents for cancer (Lungu et al., 2019). Smart biomaterials are specifically designed to be sensitive to specific stimuli present in the metastatic or tumor microenvironment (e.g., temperature, pH, etc.) and to respond by changing their structure in order to permit drug delivery, radioprotection, priming an immune response, or other functions acting as cancer therapy or enhancing its efficacy (Anderson, 2004; Mieszawska and Kaplan, 2010; Stuart et al., 2010; Mura et al., 2013). With the improvement of the fabrication techniques toward fine control of the chemical composition, multi-scale porous architecture, mechanical performance, as well as degradability, permeability and biological properties, these 4^th^ generation biomaterials have great potential in repairing bone defects induced by BM or by other degenerative diseases that impair bone cells metabolism as well as killing residual tumor cells after surgery (Ma et al., 2018). Although there are still major drawbacks to overcome, the design and development of multifunctional biomaterials matching therapy and regeneration are significantly growing; nevertheless, comprehensive research in materials science and cellular biology is still needed.

**FIGURE 2:**
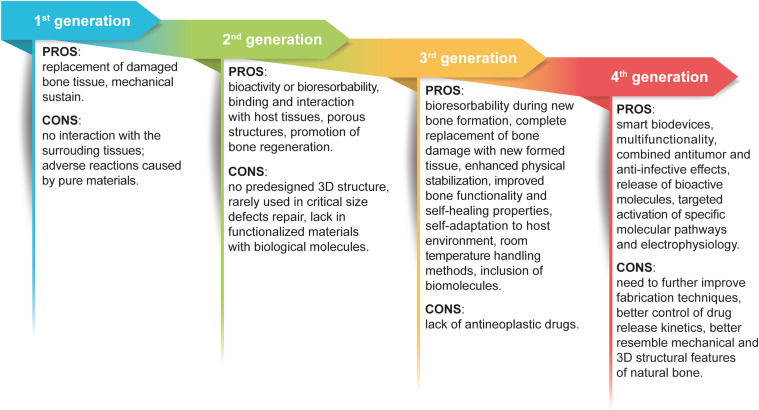
Graphic view of the evolution of biomaterials generations. The scheme highlights the pros and cons that guided the improvement of biomaterials used for orthopedic interventions and BM treatment.

For the readers’ convenience, the evolution of materials is presented as a schematic image highlighting the main pros and cons for each materials generation in Figure 2.

### Other Promising and Recently Patented Devices

As previously underlined, bone is a natural example of a composite material and today there are significant indications that taking inspiration from nature is a promising approach for the design of successful biomaterials for bone regeneration. With this concept in mind, the following paragraph describes some examples of promising composite structures; in particular, recent patented devices, methods and structures have been analyzed to provide up-to-date and tangible options for orthopedics.

Clineff et al. (2010) by Orthovita Inc. invented a bioactive composite made by PEEK and a BG, which can be used to repair, fuse and substitute damaged bones and the method to produce it. As described before, the advantages of using PEEK as polymeric base rely on its good physical and mechanical properties (e.g., 3.6 GPa Young’s Modulus, 100 MPa tensile strength, high thermal stability etc.) along with the fact that it can be molded in several forms. On the other hand, PEEK is not bioactive or osteoconductive; for this reason, inventors combined PEEK with up to 60% in weight of combeite (calcium silicate) glass-ceramic in order to improve the bioactivity of the final material. The final product is extruded and the obtained pellets can be shaped via molding. Interestingly, extrusion products can be directly injected and formed through classical injection molding procedures. PEEK-BG composite can be extruded with the possibility to modify parameters such as temperature and time of mixing, through the use of BG particles of different sizes. Bioactivity *in vitro* tests demonstrated a good deposition of calcium phosphates after 3 days of immersion in simulated body fluid (SBF), as influenced by both the percentage of combeite glass in the material and its particle size. Finally, *in vivo* tests on long bones defects in sheep showed that composite implants doubled interfacial shear strength (proxy of bone-implant bonding) in respect to pure polymer implants and titanium implants as positive control. This is accordance to mechanical tests showing that the material is able to sustain up to 5 million of load-unload cycles simulating the *in vivo* environment. In addition, histological analyses reveal new bone formation near the implant, without presence of fibrous tissue. Altogether, these results indicate that the obtained PEEK-combeite composite has potential to be used for replacement of bone segments damaged by tumors metastasis, such as spine or long bones (femur).

More recently, Teoh et al. (2019) (Osteopore International, Osteopore Korea and National University of Singapore) developed a porous (with interconnected pores) tubular bioresorbable 3D scaffold for either long bone defects or augmentation of bone length. The innovation brought by such an invention relies particularly on the production method and the organized structure of the scaffold. Inventors’ design is based on a central channel for packing/infusion of cells/tissues/bioactive molecules/drugs or a combination of them to permit osteogenesis and to preserve mechanical properties, and several side microchannels (parallel or perpendicular to the axis) permitting the formation and intercommunication of blood vessels and providing good torsional-bending strength. The patent considers several materials that are suitable for the scaffold production, such as natural and synthetic polymers (e.g., PLA, PLGA, poly(hydroxybutyrate-co-valerate) [PHB-coV], PCL) alone or in combination and suggests as preferred embodiment the production of PCL meshes spaced at least of 200 μm with the possibility to tune the porosity pattern changing the extrusion angle. The described method allows one to obtain at least 60% and up to 80% of porosity without invalidating mechanical properties, and maintaining a compressive modulus between 200 and 500 MPa. Inventors presented an example where a PCL-TCP 80:20 scaffold is produced through fused deposition modeling (FDM) extrusion. Scaffolds are 10 mm high and 4 mm in diameter, with layering pattern of 0/60/120 degrees and pore size of 500 μm; regarding mechanical properties, scaffolds present a modulus strength around 23 MPa and a compressive modulus of around 6 MPa. For *in vivo* experiments, authors tested the described scaffold in a rabbit ulnar critical size defect model dividing the animals in three groups in order to analyze different combinations of the invented device with bioactive molecules and cells. The three groups consisted in PCP-TCP scaffold only (group 1), PCP-TCP enriched with platelet rich plasma (PRP) (group 2) and PCP-TCP-PRP with the addition of MSCs (group 3). Generally, for all the evaluated parameters, such as bone mineral density, trabecular thickness and separation distribution, and bone volume fraction, all groups reached good results with the most promising ones obtained by group 3. In conclusion, the designed scaffold is able to completely integrate with surrounding tissues, forming new bone with suitable mechanical properties. Other studies or clinical trials are necessary to confirm its potential use in human long bone replacement applications.

In the context of biodegradable polymer-based composites, Volova and Shishatskaya show the construction of a composite material based on polyhydroxyalkanoates (PHAs) and calcium phosphates intended for use in the repair of bone defects of various etiologies (applicable also for BM) (Volova and Shishatskaya, 2011). The patented material is composed by a blend of 3-hydroxybutyrate (3-PHB) and 3-hydroxyvalerate (3-PHV) at varying percentage 65–90%, with a mixture of TCPs and HA from 10–35%. The structure is equipped with 20–80% of porosity (pore size 400–600 μm) obtained through the addition of various amount of sucrose crystals/sodium chloride. To increase mechanical strength, inventors added ultra-thin fibers of 3-PHB/3-PHV with a diameter of 1–3 μm; in addition, to further improve osteoinductive potential, they added a solution of BMP-2. Furthermore, the material contains 1–5% of antibiotic solution. The described material can be obtained through different techniques and in several forms such as powder, film, plates, bulk or porous scaffold, and can be used both as bone substitute or as bone filler. The *in vivo* results on mice show very good ossification and organization of mature cortical bone in all the analyzed conditions respect to blood clot control; this material is suitable for further *in vivo* studies on larger animal models to deeply demonstrate its potential use for osteo-substituting implants and/or drug delivery devices (Volova and Shishatskaya, 2011).

Generally, the design of composite materials represents the most reliable choice to set up and produce implants with the potential to show excellent *in vivo* performances. In fact, as previously anticipated, the chances that a pure material has suitable properties to be used as unique implants’ constituent are very low. For this reason, the combination of diverse materials’ species, in different forms, such as nanoparticles/fibers/tubes or 3D porous structures, and with distinct functions like fillers or coatings, represents the most successful strategy to develop functional and clinically relevant bioimplants.

## Conclusion

From the analysis of current available literature and patents, it is evident that there are some fundamental parameters that need to be considered to design an efficient implant for bone applications and, more specifically, for BM treatment in which the functionalization of the material itself with chemotherapeutics and agents to stimulate bone regeneration may be important. First, the implant should be structured in a 3D pattern with a possible hierarchical organization similar to that of bone, containing a gradient of porosity to allow the colonization, adhesion, proliferation and differentiation of healthy bone cells together with endothelial cells to permit vascularization, nutrients supply and waste removal. Moreover, the surface of materials should be designed to have adequate roughness as well as physical and chemical properties to allow cell adhesion and enhance the implant’s bioactivity, considering the chance of binding bioactive molecules or drugs (e.g., chemotherapeutic in BM patients to remove any residual cancer cells after surgery). The third fundamental characteristic is biodegradability, that should be adequately balanced with new healthy bone formation in order to avoid stress shielding and guarantee the complete substitution of the implant without the formation of toxic waste products. Furthermore, mechanical properties should be suitable for the desired application, for example load-bearing capability in femur or spine substitutions, friction resistance for joint implants and general sufficient durability and elasticity to resemble the most humanly possible bone features. Moreover, in the “race for the surface” contest with bacteria, the ideal material should avoid, at least for the first 2 weeks, bacterial biofilms formation allowing the adhesion and proliferation of patients’ fibroblasts and, therefore, bone regeneration. Several chemical and physical strategies of surface modification have been attempted and improved to obtain bioimplants being both not-infective and able to recruit healthy fibroblast to promote bone regeneration. However, this area still needs further laboratory and clinical research (Busscher et al., 2012; Neoh et al., 2012; Pham et al., 2016). Moreover, in the era of third and fourth generations of materials, printability and piezoelectricity may represent interesting properties to design and produce even more accurate custom-made personalized bioimplants with the potential to be a curative and definitive option for BM patients.

In conclusion, it is evident that there is an increasing need of interconnection and collaboration between professionals of different research areas, from biologist, biotechnologists, biomedical engineers, materials scientists, chemists and orthopedic surgeons in order to combine scientific progress and clinical needs to reach a concrete and tangible goal that could greatly benefit the longevity and quality of life for BM patients.

## Author Contributions

ANG and EL developed the concept and the design, drafted the manuscript and the figure. MM, SS, RL, LM, AT, and DD wrote, reviewed, and revised the manuscript. All the authors have read and agreed to the published version of the manuscript.

## Conflict of Interest

The authors declare that the research was conducted in the absence of any commercial or financial relationships that could be construed as a potential conflict of interest.

## Abbreviations

BC: breast cancer
BGs: bioglasses
BM: bone metastasis
BMPs: bone morphogenetic proteins
BP: bone pain
BTTs: bone-targeted therapies
β-TCP: β-tricalcium phosphate
CFS: CuFeSe2
CHA: carbonate hydroxyapatite
CNTs: carbon nanotubes
EMT: epithelial-mesenchymal transition
ECM: extra cellular matrix
FDM: fused deposition modeling
HA: hydroxyapatite
HCA: hydroxycarbonateapatite
HHM: humoral hypercalcemia of malignancy
HPPs: high-performance polymers
GO: graphene oxide
MSCs: mesenchymal stem cells
NiTi: nitinol
PHAs: polyhydroxyalkanoates
PAEK: polyaryletherketones
PEEK: poly(etheretherketone)
PEKK: poly(etherketoneketone)
PFs: pathological fractures
PGA: poly(glycolic acid)
PLA: poly(lactic acid)
PLGA: poly(lactic- co-glycolic acid)
PCL: poly(ε-caprolactone)
PHB-coV: poly(hydroxybutyrate-co-valerate)
PRP: platelet rich plasma
PMN: premetastatic niche
PMMA: poly(methyl methacrylate)
QoL: quality of life
PEMF: pulsed electromagnetic field
rhBMP2: recombinant human bone morphogenetic protein 2
RT: radiation therapy
SBF: simulated body fluid
SSt: stainless steel
SREs: skeletal related events
SSC: spinal cord compression
Ti: titanium
UHMWPE: ultrahigh-molecular-weight-polyethylene
3-PHB: 3-hydroxybutyrate
3-PHV: 3-hydroxyvalerate.
